# Editorial: Machine Learning Advanced Dynamic Omics Data Analysis for Precision Medicine

**DOI:** 10.3389/fgene.2019.01343

**Published:** 2020-02-04

**Authors:** Tao Zeng, Tao Huang, Chuan Lu

**Affiliations:** ^1^Key Laboratory of Systems Biology, Institute of Biochemistry and Cell Biology, Chinese Academy of Sciences, Shanghai, China; ^2^Shanghai Research Center for Brain Science and Brain-Inspired Intelligence, Shanghai, China; ^3^Shanghai Institute of Nutrition and Health, Shanghai Institutes for Biological Sciences (CAS), Shanghai, China; ^4^Department of Computer Science, Aberystwyth University, Aberystwyth, United Kingdom

**Keywords:** machine learning, dynamic, OMICS data, precision medicine, integration

By utilizing high-throughput technologies, precision medicine is being developed as a preventative, diagnostic and treatment tool to combat complex human diseases. It is therefore necessary to investigate how to integrate these multi-scale ‘omics datasets to distinguish the novel individual-specific disease causes from conventional cohort-common disease causes. Currently, machine learning plays an important role in biological and biomedical research, especially in the analysis of big ‘omics data. This Research Topic focuses on the application of wet ‘omics technology and dry machine learning approaches together to further develop precision medicine.

## Studies Based on Individual Temporal ‘Omics Data From Disease Cohorts or Animal Models

Liu, R. et al. proposed a single-sample-based hidden Markov model approach to detect the dynamical differences between a normal and a pre-disease states, to detect the immediately upcoming critical transition from the pre-disease state. Lee et al. implemented a deep learning-based python package for multimodal longitudinal data integration, especially the numerical data including time series and non-time series data. Yu et al. implemented an adjusted individual-specific edge-network analysis (iENA) method when a limited number of samples from one individual are available, and made a proof-of-concept study on individual-specific disease classification based on microbiota compositional dynamics.

## Studies Based on Multiple ‘Omics Data, E.G., the Combination of Genomic, Transcriptomic, Epigenomic, or Proteomic Data for a Single Disease/Condition

Chen et al. analyzed the miRNA expression profiles in whole plasma, Extracellular Vesicle (EV) and EV-free plasma of lung cancer patients and identified several discriminative miRNAs and classification rules as potential non-invasive biomarkers by Monte-Carlo feature selection method and Repeated Incremental Pruning to Produce Error Reduction method. Liu, Z. et al. conducted a genome-wide analysis of allele-specific expression (ASE) in colorectal cancer patients, providing a systematic understanding of how ASE is implicated in both tumor and normal tissues. Hu et al. used RNA sequencing data to identify and quantify the circRNAs in atrial fibrillation (AF) by bioinformatics analysis and characterized their potential functions through the competing endogenous RNA network and protein-protein interaction network. Shi, X. et al. screened a cohort of Total anomalous pulmonary venous connection cases and healthy controls for rare copy number variants by whole exome sequencing, providing candidate genes associated with rare congenital birth defect. Wu et al. performed whole exome sequencing on seven members of an HSCR family, making a first report on the in-frameshift variant p.Phe147del in RET responsible for heritable HSCR. Xie et al. investigated rare Copy number variants (CNVs) in a recruited cohort of unrelated patients with pulmonary atresia and a population-matched control cohort of healthy children by whole-exome sequencing, helping elucidate critical disease genes and new insights of pathogenesis. Meng et al. made a brief research report on the driver gene mutations in Chinese patients with non-small cell lung cancer by target sequencing and Hotspot3D computational approach together.

Ho et al. provided a review of polygenic risk scoring and machine learning in complex disease risk prediction with tissue-specific targets, expecting their power to manage complex diseases for customized preventive interventions. Li et al. identified target genes at Juvenile idiopathic arthritis risk loci in neutrophils by an integrated multi-omics approach, constructing a protein-protein interaction network on the basis of a machine learning approach. Dai et al. applied the mega-analysis of Odds Ratio (MegaOR) method to prioritize candidate genes of Crohn's Disease, based on a comprehensive collected multi-dimensional data. Wang, C.H. et al. detected differentially expressed lncRNAs and mRNAs in atherosclerosis by analyzing public datasets with the weighted gene co-expression network analysis, and this bioinformatics study would provide potential novel therapeutic and prognostic targets for atherosclerosis. Jiang, S. et al. collected and profiled the circRNA expressions of heart tissues from Atrial fibrillation patients and healthy controls, providing new insights of the circRNA roles in AF with highly potential interaction mechanisms among circRNAs, microRNAs, and mRNAs.

Gu et al. reused the Surveillance, Epidemiology, and End Results registry database to conduct stratification analyses, univariable and multivariable analyses, indicating surgery is an important component of multidisciplinary treatment and sublober resection is not inferior to lobectomy for the specific patients. Zhang, J. et al. exploited the largest crohn's disease dataset and ulcerative colitis dataset by a two-step approach, exhaustively searching for epistasis with dense markers and exploiting marker dependencies. Du et al. analyzed the genome-wide splicing data in 16 cancer types with normal samples by a network-based and modularized approach and captured the pan-cancer splicing and modularized perturbation, which support the dominant patterns of cancer-associated splicing. Zhao et al. assessed the prognostic value of Apolipoprotein E and explored the potential relationship with tumor progression in colorectal cancer (CRC), by collecting the microarray data from the Gene Expression Omnibus and exploring the gene with prognostic significance from the TCGA database. Tang et al. proposed an effective data integration framework HCI (High-order Correlation Integration) to realize high-dimensional data feature extraction with extensive flexibility and applicability on sample clustering with RNA-seq data on bulk and single-cell levels. Chang et al. identified new susceptibility genes and causal sub-networks in schizophrenia by an integrated network-based approach, and reported the N-methyl-D-aspartate receptor interactome highly targeted by multiple types of genetic risk factors. Wang and Liu recognized potential diagnostic biomarkers of Alzheimer's disease by integrating gene expression profiles from six brain regions in a machine-learning manner and validating marker genes in multiple cross-validations and functional enrichment analyses. Xu et al. provided an effective way for the annotation of nuclear non-coding and mitochondrial genes and the identification of new steady RNAs, making a pan RNA-seq analysis to suggest the ubiquitous existence of both 5' and 3' end small RNAs.

## Studies Based on the Gut Metagenome and Host ‘Omics for Complex Diseases Diagnosis and Treatment

Yang et al. presented a new pathogen detection and strain typing method UltraStrain for Salmonella enterica based on whole genome sequencing data, which includes a noise filtering step, a strains identification step on the basis of statistical learning, and a final refinement step. Tan et al. conducted comprehensive and systematic experiments, including *in vitro* genetic assessments and an *in vivo* acute toxicity study, aiming to study safety issues associated with Bacteroides ovatus ELH-B2. Qiu et al. set up an in-silico model emerging or re-emerging dengue virus (DENV) based on possible antigenicity-dominant positions of envelope (E) protein, so that, the DENV serotyping may be re-considered antigenetically rather than genetically. Zhang, B. et al. collected and re-analyzed the published fecal 16S rDNA sequencing datasets to identify biomarkers to classify and predict colorectal tumors by random forest method, and the trained random forest model has good AUC performance for CRC when combined all samples, although the predication performed poorly for advance adenoma and adenoma.

## Studies Based on Conditional Genotype-Phenotype Detection With Deep Learning or Other Brain-Like Artificial Intelligence (AI) Technologies

Luo et al. proposed a manifold learning-based method to predict disease-gene associations by assuming that the geodesic distance of related disease-gene pairs should be shorter than that of non-associated disease-gene pairs. Tkachev et al. proposed a heuristic technique termed FLOating Window Projective Separator (FloWPS) for data trimming with SVM and applied it for personalized predictions based on molecular data. Wang, W. et al. developed a new multiple-instance leaning algorithm derived from AdaBoost and accessed this algorithm on annotating proteins that bind DNA and RNA. Xiao et al. proposed a method called BPLLDA to predict lncRNA-disease associations from a heterogeneous lncRNA-disease association network assuming the association paths on network with fixed lengths. Zou et al. used decision tree, random forest and neural network to predict diabetes mellitus by the hospital physical examination data, and the best prediction could be achieved by random forest after dimensionality reduction by principal component analysis and minimum redundancy maximum relevance.

Guo et al. proposed a new approach SGL-LMM for mining multivariate associations of quantitative traits by combining sparse group lasso and linear mixed model together, which can consider confounding effects and groups of SNPs simultaneously. Zhang, W. et al. developed a new calling method for differentially expressed genes as DECtp by integrating tumor purity information into a generalized least square procedure and a follow-up Wald test. Cheng et al. utilized a Mendelian randomization (MR) to test the influence of body mass index (BMI) on the risk of T2DM based on GWAS data, validating the causal effect of high BMI on the risk of T2DM. Feng et al. utilized one analysis procedure of feature selection and classification on both transcriptomes and methylomes cancer data, suggesting age should be an essential factor rather than confounding factor in the training and optimization of disease diagnosis model.

Qin et al. developed a new joint gene set analysis statistical framework, aiming to improve the power of identifying enriched gene sets by integrating multiple similar disease datasets when the sample size is limited. Shi, Q. et al. proposed a new computational framework of “Multi-view Subspace Clustering Analysis” to capture the underlying heterogeneity of samples from multiple data types, by first measuring the local similarities of samples in the same subspace and then extracting the global consensus sample patterns. Jiang, P. et al. developed a new variants mining algorithm based on trio-based sequencing data, and applied this method on a Ventricular septal defect (VSD) trio and identified several genes and lncRNA highly related to VSD.

Finally, we sincerely thank the reviewers for their great efforts to ensure the high quality of all contributing articles, and we hope this Research Topic can attract wide attention in these topics of precision medicine based on machine learning and omics data.

## Author Contributions

TZ drafted the manuscript. TZ, TH, and CL revised the manuscript.

## Funding

This study was supported by the National Natural Science Foundation of China (11871456), the Shanghai Municipal Science and Technology Major Project (2017SHZDZX01), and the Natural Science Foundation of Shanghai (17ZR1446100).

## Conflict of Interest

The authors declare that the research was conducted in the absence of any commercial or financial relationships that could be construed as a potential conflict of interest.

